# Artificial intelligence in cancer immunotherapy: current trends in predicting response and personalizing treatment

**DOI:** 10.1186/s43046-026-00371-w

**Published:** 2026-05-28

**Authors:** Eloghosa Aisosa Nosa-Ihaza, Emmanuel Chidera Edeh, Wol Bol Geng, Ebenezer Okenwa

**Affiliations:** Department of Pharmacy, Marwadi University, Rajkot, India

**Keywords:** Cancer immunotherapy, Artificial intelligence, Treatment response prediction, Biomarker discovery, Tumor microenvironment, Precision oncology

## Abstract

Artificial intelligence (AI) can transform cancer immunotherapy by enabling more accurate prediction of treatment responses, the discovery of specific biomarkers, and the development of personalised treatment plans. Traditional single-marker biomarkers (PD-L1, TMB, MSI) lack consistency across tumour types and cannot be used to assess tumour heterogeneity or the dynamic tumour microenvironment (TME). This review synthesises developments in multimodal AI models that combine genomics, transcriptomics, radiomics, digital pathology (pathomics), circulating biomarkers, and clinical evidence to create composite predictive signatures with significantly better discriminatory value. AUCs over 0.8 have been seen in a few retrospective studies with deep learning and ensemble models on whole-slide images, CT/MRI/PET radiomics, spatial and single-cell omics, and multi-omics fusion models, but prospective and multicentre validation is scarce, and external validation often shows deterioration in performance. AI is also used to enhance the translational pipelines of adoptive cell therapies (e.g., CAR-T) by improving patient selection, manufacturing (e.g., digital twins), and early toxicity prediction (e.g., CRS, ICANS). Nevertheless, clinical implementation remains hindered by data heterogeneity, bias, poor longitudinal validation, limited reproducibility, and a lack of transparency in most models, even though prospective, multicenter validation and explainable AI are crucial for clinician trust and regulatory acceptance. New systems such as federated learning, foundation models, spatial omics, digital twins, and wearable monitoring represent paths to generalizable, privacy-preserving, and actionable systems in clinical practice. To achieve the potential of AI, the generation of data will need to be standardized, reporting must be transparent, interdisciplinary, and regulatory frameworks must be strengthened focusing on the practical use of AI and patient safety. By taking these steps, AI could be shifted to prospective clinical decision support, which uses AI to meaningfully enhance personalization and outcomes in cancer immunotherapy based on a retrospective research tool.

**Graphical abstract:**

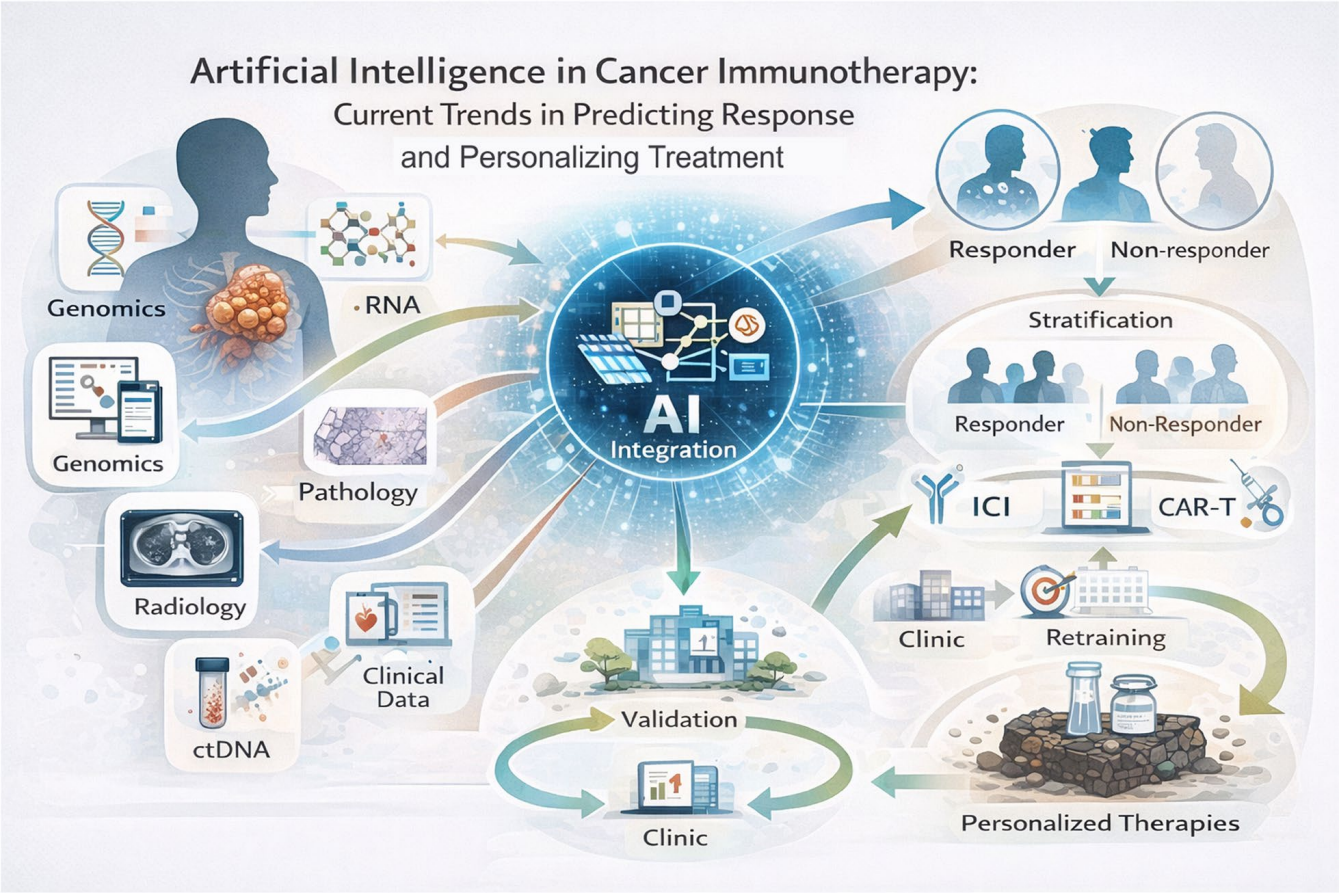

## Introduction

Immunotherapy has transformed oncology into a relatively reliable, sometimes surgery-sparing response [1, 2]. Several approaches, including cancer vaccines, monoclonal antibodies, oncolytic viruses, cytokine therapies, and immune checkpoint inhibitors, have revolutionised treatment for both solid and haematological malignancies [3]. Thus, patients and therapists gain justifiable optimism about survival chances and quality of life [4]. This is especially true in metastatic cases that were previously considered untreatable [5]. Several research projects have identified the potential of CAR-Macrophages (CAR-M), a type of immune cell engineered to recognise tumours [6]. These can be used both alone and in combination with PD-1 blockade (an immune checkpoint inhibitor) to address tumour resistance to monotherapy [7]. Relatedly, breakthrough discoveries into interleukin-15 (IL-15), a signalling protein that helps regulate immune cell activity, reveal its potential to revitalise NK cells within tumours [8]. These findings restore the cancer-fighting abilities of natural killer (NK) cells, a class of immune cells that target tumour cells.

The benefits of immunotherapy are not without shortcomings. These include its cost, accessibility, risk of adverse effects, and cytotoxicity. Furthermore, cancerous growths can circumvent immunosurveillance by manipulating antigenicity and immune checkpoints [9, 10]. When this occurs, therapy resistance or relapse may result, requiring sequential or combination therapies to sustain responses. A pressing limitation of immunotherapy is the inability to predict patient response [11]. Not all patients benefit, as response rates vary due to tumour heterogeneity, genetic diversity, and the complex tumour microenvironment. Most available biomarkers, including tumour mutational load (the number of mutations in tumour DNA) and PD-L1 expression (a protein that inhibits the immune system), are only partial predictors of response [12]. These predictions are also not accurate due to several factors, such as epigenetic changes (alterations in gene activity, not the DNA sequence) and subclonal divergence in tumours (variations within groups of cells within the same tumour) [13]. Besides, most existing biomarker assays are not universally available and can miss important mutations because they rely too heavily on genomic profiling (comprehensive analysis of a tumour’s genetic material), which is very costly.

However, translating biomarker research into clinical practice is complicated by limited funding and poor integration of data sources [14]. As a result, these issues lower the reliability and clinical utility of immunotherapy. Overcoming these barriers remains a critical goal in the field [15]. AI systems in oncology are transforming the process of early detection and proper diagnosis of cancer [16]. By excelling at handling complex radiology and pathology data, they can detect elusive abnormalities early, which is a significant advantage over the traditional approach. As a result, these faster diagnoses and earlier recurrence detection can be months or years ahead of other methods.

AI models also dominate predictions of patient immunotherapy response, drawing on genomic, clinical, tumour, and blood test data [17]. Building on this, more advanced tools such as IRRS leverage multi-omics data and immune gene signatures to classify patients by therapy. Various studies confirm their accuracy in colorectal and other cancers. AI’s ability to generate and screen protein molecules quickly can equip patient immune cells with custom-made treatments [18]. By accelerating development timelines, from weeks to years, AI also guides the development of potential treatments based on an individual’s genetics, microbiome, and tumour biology. Therefore, physicians can potentially deliver cancer immunotherapy in a much shorter timeframe, with more precise predictions tailored to each patient. This integration of AI holds promise for overcoming earlier barriers to personalisation and access.

This narrative review focuses on the use of AI to predict immunotherapy response and personalise cancer treatment in solid and haematological malignancies. The literature was identified by searching PubMed, Google Scholar, and clinical trial registries through early 2025 using the following terms: artificial intelligence, machine learning, immunotherapy, biomarker prediction, tumour microenvironment, CAR-T, and radiomics. Original research studies, prospective or externally validated analyses, and landmark reviews when primary studies were not available were given priority. The modalities of AI include genomics- and transcriptomics-based models, radiomics, digital pathology, multi-omics integration, and emerging modalities such as federated learning, foundation models, spatial omics, and digital twins.

## Landscape of immunotherapy response

Even though immunotherapy has proven to be clinically effective in several cancer types, predicting responses in patients remains unresolved. Others can achieve lasting remission, while others gain little or no benefit, and this difference can be attributed to a complex interaction among tumour heterogeneity, epigenetic modifications, and a dynamic tumour microenvironment [19, 20]. Common examples of biomarkers, such as PD-L1 expression, are promising but inconsistent in their predictive power and not universally available, which is why more complex, integrative predictive systems are needed.

### Limitations of current biomarkers

The existing biomarkers, including PD-L1 expression and microsatellite instability (MSI), have no reliable predictive value for immunotherapy response [21]. They are limited by their failure to select patients and treat them properly. Consequently, tailored treatment advice remains suboptimal. Below are some of these key restrictions:

- Inconsistent predictive value: Unfortunately, PD-L1 and TMB give variable responses across cancer types. These biomarkers, whether high or low, are not foolproof predictors of immunotherapy success, as patients in either group may not respond to treatment [22].
- Intratumoral and Patient Heterogeneity: There is no uniformity in biomarker expression across tumour regions, and this heterogeneity may vary over time. Therefore, a single biopsy sample cannot represent the biology of a whole tumour. The diversity of subclonal neoantigens (distinct proteins arising from mutations in tumour cells) also makes prediction more difficult, since the clones that initiate immune evasion may persist even with high biomarker levels [23].
- Lack of Standardisation: Testing platforms for biomarkers differ markedly in methods, sample requirements, and scoring cutoffs. These promote inconsistent and contradictory results across laboratories and clinical settings [24].
- Complex and Unpredictable Toxicity: Currently, biomarkers are unreliable for predicting or preventing immune-related adverse events. They complicate patient management and therapy selection, forcing physicians to consider other treatment options [25].

The majority of data on biomarkers to support immunotherapy remains investigational. These biomarkers require large, successful clinical trials to be validated and gain acceptance.

**Fig. 1:**
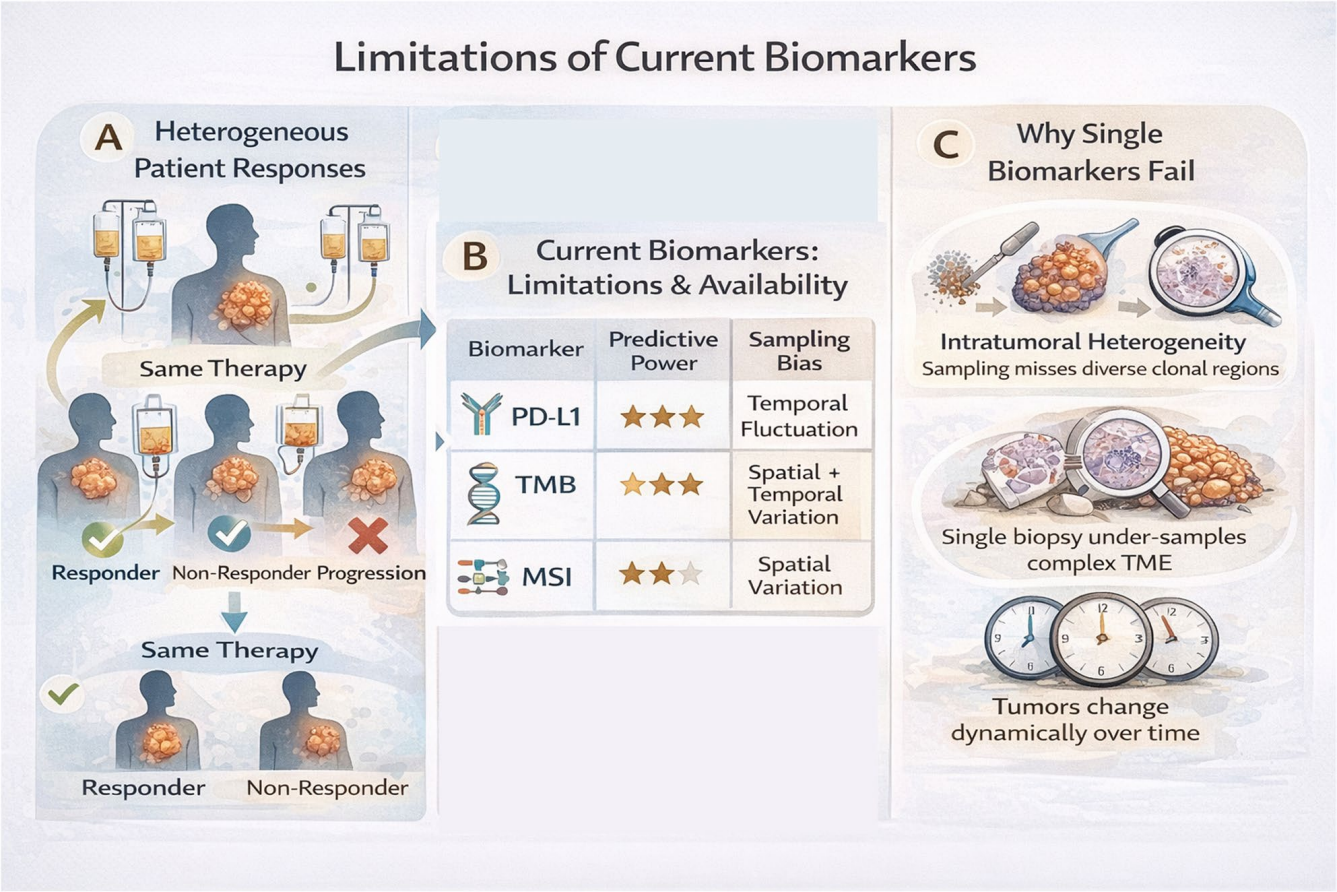
Limitations of current biomarkers in predicting immunotherapy response. **A** Heterogeneous patient outcomes, including responders, non-responders, and progressors, arising from the same immunotherapy regimen, illustrating the fundamental challenge of response prediction. **B** Comparison of PD-L1, TMB, and MSI across predictive power and sampling bias dimensions, highlighting the temporal and spatial variability that limits their clinical utility. **C** Key reasons why single-biomarker approaches fail: intratumoral heterogeneity causes biopsy under-sampling of diverse clonal regions; single-site biopsy cannot capture the full complexity of the tumour microenvironment; and tumours change dynamically over time, rendering static biomarker snapshots insufficient. TMB, tumour mutational burden; MSI, microsatellite instability; TME, tumour microenvironment

Figure 1 illustrates the key limitations of current biomarker-based immunotherapy prediction approaches.

### Complexity of the tumour microenvironment (TME)

The tumour microenvironment (TME) is a complex living environment. It contains tumour cells, immune cells that defend the body, stromal cells that support the body, endothelial cells that line blood vessels, and the extracellular matrix, a system of molecules outside cells. It has been found that the TME elements mediate cancer metastasis, immune homeostasis, and immune response to treatment via intricate signalling pathways and metabolic networks [26]. TME components comprise T cells (immune cells that attack infected or cancerous cells), regulatory T cells (Tregs, which suppress immune responses), myeloid-derived suppressor cells (MDSCs, which inhibit immune function), and tumour-associated macrophages (TAMs, immune cells within tumours that can support or suppress immune responses). These cells generate cytokines (protein-based cell signalling), metabolites, and growth factors that inhibit antitumor mechanisms, activate immune suppression, and stimulate tumour development [26].

The extracellular matrix and abnormal vasculature surrounding tumour cells shape the tumour’s structure. They also serve as barriers to immune cell infiltration and weaken immunotherapy response. The TME’s metabolic reprogramming leads to nutrient starvation and immunosuppression of metabolites. This triggers tumour resistance to immune checkpoint inhibitors [27]. Immune evasion is promoted within immunosuppressive networks, including chemokine signalling and the complement system [28]. The entry of cytotoxic lymphocytes is blocked by physical barriers, such as angiogenesis and the dense production of matrices. These barriers limit the effectiveness of immunotherapy.

### Rise of integrated/multi-modal biomarkers

Combination or multi-modal biomarkers, a collection of different streams of data (genetic, imaging, and clinical data), are quickly becoming a game-changer in immunotherapy response prediction. These data modalities, including genomics (DNA information), transcriptomics (gene activity data), radiomics (features from medical imaging), histopathology (microscopic examination of tissues), blood biomarkers (measurable substances in blood), and clinical features, outperform conventional single-modal markers (such as PD-L1 and TMB) [29]. Single biomarkers are not accurate enough to predict immune responses. It stems mainly from their monomolecular nature; as such, responses driven by complex, dynamic interactions demand a more robust solution. On the other hand, integrated models broaden the view of tumour biology, immune status, and treatment response. They reflect the varied mechanisms of immune escape and resistance [29].

For example, deep learning frameworks, such as deepAFM, integrate various types of data, including histopathology, genomic, radiomic, and clinical data, to improve predictions of immunotherapy response. Studies have reported AUC values up to 0.80, as it also identifies key patterns and features associated with positive outcomes [30]. Integrating multi-omics with radiomics and pathomics yields more robust predictive signatures than using any single modality alone. Combining imaging features and digital pathology with molecular biomarkers gives better prognostic information than each feature alone.

Results for ctDNA dynamics, tumour tissue-based RNA profiles, and clinical variables demonstrate improved sensitivity and specificity for predicting the benefit from immune checkpoint inhibitors compared with single-platform approaches [30].

Table 1 summarises the predictive accuracy, availability, standardisation, and clinical utility limitations of the three most widely used conventional immunotherapy biomarkers: PD-L1, TMB, and MSI [29, 30].

**Table 1:** Limitations of conventional immunotherapy biomarkers (PD-L1, TMB, MSI) across key dimensions [29, 30]

Biomarker	Predictive accuracy	Availability	Consistency / Standardization	Clinical utility
PD-L1 expression (IHC)	Highly variable; not a foolproof predictor	Widely available via IHC in most oncology laboratories	Low (assays and cutoffs vary between laboratories)	Used for patient selection in some cancers (e.g., lung, breast), but many false-negative/positives
Tumor mutational burden (TMB)	Variable; only a partial predictor	Requires NGS panels (whole-exome or targeted); costly and not universal	Low (no universal threshold; values depend on panel size and calling method)	FDA-approved for pembrolizumab in TMB-high tumors; modest incremental predictive value
Microsatellite instability (MSI) (dMMR)	High predictor in MSI-H tumors	Routine PCR/IHC testing in colorectal and select other cancers (per guidelines)	High (standardized testing methods; MSI-H reliably detected)	Approved as a pan-cancer ICI biomarker; limited by low prevalence (~ 5%)

*Notes: *IHC* immunohistochemistry (antibody clones and scoring schemes vary), *mut/Mb* mutations per megabase

## AI models and data modalities

Immunotherapy AI uses patient data (including genetic information, lab results, images, and health records) to identify treatment responders. The integration of various data sets helps identify intricate biomarkers and predict tumour-immune responses, using AI to support doctors in selecting the most effective treatment for an individual [29, 30].

### Genomics and transcriptomics

Genomics and transcriptomics offer a high-dimensional perspective on tumour biology: genomics describes the mutational landscape, and transcriptomics describes dynamic gene expression patterns across cell types and microenvironmental states. AI integrated with bioinformatics and multiomics (e.g., genomics, transcriptomics) reveals tumour evolution, identifies useful biomarkers, and supports drug selection. Emerging tools, such as the Deep DR model for genomic data, face challenges with standardisation and interoperability when used to guide immunotherapy decisions [18]. AI utilisation involves the use of large-scale genomic data to support personalised therapy, early diagnosis, prognosis, and drug development. Big data applications combine genomics and transcriptomics with deep learning to predict variants, drug response, and genetic changes, informing treatment [18].

One of them is AI-based incorporation of genomic, transcriptomic, proteomic, and epigenomic data to establish multi-omics biomarkers, model tumour behaviour, and personalise immunotherapy. For example, neural networks can predict peptide-MHC binding affinity and, as such, have the potential to identify neoantigens, while transcriptomic models can model features of the tumour microenvironment that can serve as predictors of targeted immunotherapy [29].

### Radiomics and medical imaging

AI combines radiomics with imaging techniques, which include CT, MRI, PET, and mammography, to detect lesions, stratify risk, and forecast outcomes. Deep learning uses a combination of imaging, genomic, and clinical data to enhance the diagnosis and prognosis of cancer. Genetic mutations and outcome tracking can be predicted using non-invasive biomarkers, including radiogenomic methods. There are challenges in clinical use of data standardisation, reproducibility, infrastructure, and ethics [16]. Immunomics and new imaging are used to measure immune-related side effects of immunotherapy. Using AI also improves the assessment of responses and toxicity from images, as PET/CT and other imaging techniques have advanced.

This is practised through continual monitoring, which is possible with these approaches. Combining clinical and imaging data in the oncology field enhances risk stratification and tailored planning, and radiomics-AI integration in clinical decision support. Multimodal imaging (CT, MRI, and PET/CT) is used to predict tumour behaviour, recurrence, and response using machine learning models [30]. The MRI-based radiomics clinical nomogram to predict pathologic complete response after neoadjuvant immunotherapy is significant because it highlights how AI-based radiomics can support the optimisation of personalised treatment and the selection of patients to inclusion in immunotherapy trials [16, 30].

### Digital pathology and pathomics

Artificial intelligence-based digital pathology using H&E-stained slides for immuno-oncology analyses the utility of deep learning and computational approaches on whole-slide images (WSIs) to identify and quantify immune biomarkers, stratify patients for immune checkpoint inhibitors (ICIs), and predict immunotherapy response and survival [11]. It addresses challenges such as tissue sampling, the subjectivity of manual quantification, and future directions for clinical deployment. Digital pathology-based AI models (pathomics) can analyse histopathological images to predict tumour grade, treatment response, and recurrence [29].

By applying deep learning, these models support personalised immunotherapy strategies and, when integrated with multi-omics data, can improve clinical decision-making in cancer care. A digital pathology model using AI, combined with whole-slide image validation, can predict response to immune checkpoint inhibitors, risk-stratify patients, and monitor long-term alterations in the tumour microenvironment during immunotherapy [29]. Their usefulness in explainable AI models has also been demonstrated in recent work, which shows their value for assessing model rigour, bias identification, and hypothesis-based interpretation beyond traditional attention maps [22, 26].

Immunotherapy response and prognosis in gastric cancer were accurately predicted using a multimodal radiopathomics signature (RPS), which combined digital H&E-stained pathology images with CT scans and was trained with seven machine learning models (AUC: 0.978 − 0.822 in validation cohorts). Genetic measurements also linked the RPS to immune-related pathways and to memory B-cell infiltration, providing biological interpretability and facilitating accurate decisions about immunotherapy use, respectively [29].

### Other input types

Various types of data for AI analysis are becoming standard, such as H&E stainings, IHC, gene expression, genetic changes, TCGA data, and immune cell densities, especially in colorectal cancer immuno-oncology [26]. Multimodal AI is employed to predict MSI status and immune response, as well as survival, in many studies that employ deep learning, support vector machines, and ensemble methods to obtain robust predictions. Advanced input types, such as multiplex immunohistochemistry (mIHC) and single-cell profiling, enable high-resolution mapping of immune cells in the tumour microenvironment. These methods are beyond the reach of standard H&E and can perform spatial analysis of cell interactions and model the complexity of the melanoma tumour ecosystem. The combination of these data with digital pathology and AI will improve diagnostic accuracy and enable more accurate prediction of immunotherapy response, enabling individualised treatment [29].

Table 2 provides an overview of the major AI data modalities used in immunotherapy, including their typical methods, clinical use cases, and reported predictive performance [26, 29].

**Table 2:** AI data modalities, typical methods, use cases, and performance in immunotherapy [26, 29]

Data modality	AI methods	Use cases	Reported performance
Radiomics (CT/MRI/PET imaging)	CNNs (e.g., ResNet) + ML (SVM, RF, LASSO)	Predict PD-L1 status or ICI response (e.g., NSCLC)	AUC = 0.83 for PD-L1 status (NSCLC)
Digital pathology (H&E whole-slide images)	CNNs (deep learning on WSIs)	Predict PD-L1, identify responders (breast, lung, etc.)	AUC ~ 0.92 for PD-L1 expression (breast cancer)
Multi-omics integration (histology + genomics + imaging + clinical)	Multi-input deep learning (e.g., deepAFM)	Integrate diverse data to predict response	AUC ~ 0.80 (multi-model deepAFM example)
Spatial omics (spatial transcriptomics/proteomics)	Graph neural nets / CNN on spatial maps	Map tumor-immune interactions, predict resistance features	AUC = 0.838 (NSCLC spatial multi-omics)

*CNN* convolutional neural network, *RF* random forest, *SVM* support vector machine, *WSIs* whole-slide images

## AI applications in solid tumours

The complexity of solid tumours, their heterogeneity, and the various immune evasion strategies make them a major challenge for immunotherapy. AI can help overcome these barriers by combining imaging and clinical data to track response, detect biomarkers, and customise treatment to individual patients’ needs. The following sections discuss the application in melanoma, gastrointestinal cancers, non-small cell lung cancer, and other solid tumours.

### Melanoma: the immunogenic gold standard

Melanoma has a high mutational load and is likely to respond to immunotherapy. AI leverages whole-genome and transcriptomic data to identify immunogenic mutations for immune-based therapies. Deep learning and immunogenicity scales operate at scale to accelerate the discovery and validation of the mutation genome for each patient [1]. Below are core AI applications in solid tumours:

- Neoantigen Discovery: Neoantigen candidates are typically identified using AI-based algorithms and immunoinformatics, and then used to develop cancer vaccines tailored to each patient’s unique cancer mutation landscape.
- Adoptive Cell Therapy Optimisation: Predictive models refine T cell selection and targeting for ACT, ensuring high specificity for patient-specific neoantigens and reducing adverse effects.
- Personalisation of therapy: AI can rank patients based on their anticipated response to immunotherapy, guiding clinicians to the most effective therapy and combination regimen, and monitoring resistance mechanisms.
- Radiomics and Noninvasive Diagnostics: AI-based radiomics can detect hidden cues in the imaging of diseased tissue (CT, MRI, PET) that the human eye cannot see, providing indicators of therapy response and overall survival, even in some cases without the need for tissue biopsies to detect mutations.
- Histopathology and Genomics Fusion: Models like Pathomic Fusion combine histological slide images with genomic data to enhance predictive, prognostic, and treatment-response information and are effective in tumours such as glioma and renal cell carcinoma. Equally, deep learning models have integrated histology with omics data across various cancer types, outperforming modality-specific models and providing features such as immune cell infiltration.

The application of AI to melanoma and other solid tumours demonstrates how combining genomic, imaging, and clinical data can enable highly personalised immunotherapy. By identifying a treatment approach tailored to a patient’s tumour genotype, immune microenvironment, and clinical history, this approach also accelerates biomarker discovery and therapeutic optimisation, ultimately contributing to the larger goal of precision oncology [1, 22].

### NSCLC: real-world AI implementations

NSCLC is among the most prevalent cancers globally, with substantial real-world clinical data available, making it a natural proving ground for AI applications in immunotherapy. AI models trained on clinical, radiomic, and genomic data help forecast responses to immune checkpoint inhibitors, improving prediction accuracy and enabling more personalised treatment decisions. AI plays a daily role in analysing clinical, radiomic, and genomic data to improve treatment with immune checkpoint inhibitors and more accurately predict how patients will respond than traditional methods [16].

#### Machine learning for anticipating immunotherapy response

Recent research has discovered that machine learning models trained on real-world patient data can forecast the effectiveness of immunotherapy in non-small cell lung cancer (NSCLC). CatBoost, logistic regression, neural networks, and support vector machines are among the models tested to assess their predictive capabilities for outcomes such as disease control rate, overall survival, and time to treatment failure [16]. The neutrophil-to-lymphocyte ratio, performance status, PD-L1 levels, treatment line, and status of combination therapy are all important clinical predictors [17].

#### AI tools and FDA recognition

An AI-powered prognostic tool developed by Onc.AI (Serial CTRS) recently received FDA breakthrough device designation. It is essential to utilise deep learning analysis of imaging scans from real-world datasets to differentiate NSCLC patients into high and low-risk groups during treatment. The tool’s goal is to improve the effectiveness of oncology care by enhancing risk classification. This will help oncologists make better decisions based on automated, reproducible prognostic insights [16, 17].

### GI cancers (Gastric, Colorectal): emerging challenges

Challenges such as data quality, biomarker limits, and clinical implementation are emerging in gastrointestinal (GI) malignancies. Currently, GI cancer datasets are often heterogeneous or lack standardisation, making it challenging for AI models to consistently learn patterns and generalise findings across diverse patient populations [14]. Most AI-trained models for diagnosing or predicting gastric or colorectal cancer are not externally validated. This raises questions about their reproducibility and usefulness in standard clinical practice [18]. AI model integration into clinical practice faces limitations in explainability, bias, and regulatory issues related to safety and efficacy; it is being evaluated as a factor guiding immunotherapy use regardless of its effects on other clinical factors [20, 23].

As AI applications move beyond research into the clinic, ethical and legal concerns such as data privacy, algorithmic transparency, and equitable access continue to be the subject of discussion [20]. Therefore, AI has tremendous potential in enhancing the use of GI cancer immunotherapy; these emerging issues need to be properly systematised to facilitate safe, effective, and equitable implementation in the clinic [21, 28].

### Other solid tumours (e.g., Breast, Glioblastoma)

In breast cancer, primarily in the triple-negative form of the disease, AI is undergoing evaluation as a predictor of immune biomarkers and to separate patients who are most likely to respond to immunotherapy, despite shared unresponsiveness [11]. Glioblastoma can be challenging due to its immunosuppressive microenvironment; in this context, AI helps analyse tumour heterogeneity and immune escape mechanisms, and identify potential targets. The same strategy is being applied to renal cell carcinoma and head-and-neck cancer, where AI analyses pathology information alongside imaging and other molecular data to decide whether an individual should receive immunotherapy [26]. Together, these applications underscore the potential of AI to expand personalised immunotherapy to non-metastatic solid tumours that are historically immune-resistant. By improving the predictive capacity of immunotherapy, diagnosis, and tailored treatment plans, AI is redefining the management of solid tumours, including breast cancer and glioblastoma.

#### Breast cancer

AI combines complex biomarker data (PD-L1, TMB, TILs, MSI) to predict immunotherapy response. This guides the selection of patients most likely to benefit from immune checkpoint inhibitors [22]. When used together (genomics, transcriptomics, and imagery), AI would enable individualised predictions of immunotherapy effectiveness, particularly in difficult-to-treat subtypes such as triple-negative breast cancer [29]. Standardising and automating the scoring of immune biomarkers and tumour-infiltrating lymphocytes using AI-based pathology tools makes these tools more accurate, less prone to observer bias, and better suited for individualised therapy through imaging and molecular profile analysis, as well as for predicting response to therapy [11].

#### Glioblastoma

In glioblastoma, AI can aid immunotherapy through imaging and molecular profiling, predicting therapy response, simulating therapy response, and identifying potential resistance mechanisms [26]. AI enables combining longitudinal clinical and molecular data to simulate tumour evolution and immune evasion, a significant obstacle in this heterogeneous and aggressive tumour. Multi-modal AI models have been investigated to better stratify experimental immunotherapies; however, significant challenges persist due to insufficient data and the unique microenvironment of brain tumours [30]. AI is leading the way in creating tailored immunotherapy for breast cancer and glioblastoma. However, further validation is still needed to fully realise its potential.

## AI applications in haematological malignancies

### CAR-T cell therapy: predictive modelling and manufacturing optimisation

CAR-T cell therapy represents one of the most significant breakthroughs in cancer immunotherapy [6]. The incorporation of artificial intelligence (AI) into CAR-T therapy has transformed the approach to challenges across patient selection, personalised treatment, and improved manufacturing [29]. This analysis reviews current applications, limitations, and the future outlook of AI-driven predictive modelling in manufacturing CAR-T cell therapy.

#### Biomarker discovery and patient selection

AI has revolutionised the identification of predictive biomarkers in CAR-T therapy, enabling the accurate prediction of treatment outcome through advanced machine learning models [29]. AI-powered multi-omics integration enables the analysis of genomic, proteomic, and metabolic data to profile patients in detail [30]. Biomarkers of CAR-T therapy identify failures using models such as InflaMix, providing more accurate information than clinical markers.

#### Adverse event prediction

One of the most critical applications of AI in CAR-T therapy is the detection of adverse effects. Machine learning models accurately predict cytokine release syndrome using real-time patient data (AUC up to 0.90) and warn of its onset up to 72 h before clinical manifestation [25]. Similarly, neuroimaging and clinical models for ICANS accurately identify high-risk patients for early interventions to improve patient safety and outcomes [27].

#### Manufacturing optimisation through digital technologies: digital twin implementation

A digital twin is a powerful tool that is revolutionising CAR-T manufacturing by enabling the creation of virtual models of the production process, as demonstrated by multiple use cases in the AIDPATH project [28].

1. *Real-time cell expansion monitoring*: Digital twins predict optimal CAR-T cell harvest by modelling growth based on nutrient and metabolite levels [28].
2. *Soft sensor integration*: An AI-powered sensor network interprets bioreactor data to provide real-time alerts and optimisation recommendations [28].
3. *Production scheduling optimisation*: Machine learning algorithms synchronise multi-patient manufacturing and manage uncertainties in cell expansion timeline [28].

### AI in predicting response/toxicity in lymphomas and leukaemias

The prediction of therapeutic responses and toxicity in leukaemia and lymphomas is advancing with Artificial Intelligence (AI), enabling precision medicine for blood cancers.

#### Predicting treatment response

1. *Imaging-Based Models*: The imaging characteristics of AI-driven PET/CT scans are accurate predictors of lymphoma and treatment response, and outperform conventional metrics [11]. In one example, ibrutinib response predicts the use of pre-therapy models with an AUC value up to 0.86 [18].
2. *Multi-Modal and Genomic AI*: The combination of clinical, genetic, pathology, and imaging information in AI models to personalise relapse and survival predictions in lymphoma is more effective than conventional risk scores [29]. Explainable AI and decision trees categorise patients with Hodgkin lymphoma to make the most effective treatment [29].
3. *CAR-T and Immunotherapy*: Based on deep learning, AI-based tests forecast lymphoma patient outcomes with immunotherapies such as CAR-T, through patient-specific treatment planning [11, 29].

#### Predicting and Monitoring Toxicity

1. *AI for Adverse Event Risk*: Machine learning algorithms can forecast severe immunotherapy toxicities, such as CRS and neurotoxicity, particularly during CAR-T therapy for leukaemia and lymphoma [25].
2. *Psychosocial Outcomes*: This use of AI to analyse patient-reported outcomes has revealed groups of lymphoma and leukaemia patients who are at high risk of experiencing severe psychosocial toxicity, facilitating more comprehensive care [27].

Table 3 summarises the three major categories of AI models used for predicting and monitoring immunotherapy toxicity, their target outcomes, cancer contexts, and reported performance metrics [11, 19, 20, 23, 25, 27].

#### Limitations and future directions

1. *Data Standardisation & Multicenter Validation*: Heterogeneous datasets and variable clinical workflows hinder the effectiveness of AI tools [19].
2. *Algorithm Interpretability*: Decision trees and explainable AI models are gaining popularity to build clinician trust and facilitate regulatory compliance [23].
3. *Integration into Care*: The use of AI-based predictive models will have the most significant impact when integrated with clinical decision support systems, facilitating adaptive therapy that leverages real-time patient data [20].

**Table 3:** AI models for predicting and monitoring immunotherapy toxicity [11, 19, 20, 23, 25, 27]

AI model/data	Target toxicity outcome	Cancer context	Reported performance
ML on clinical data (e.g., RF, XGBoost)	Cytokine release syndrome (CRS) and neurotoxicity (ICANS)	CAR-T therapy (B-cell leukemias/lymphomas)	AUC up to 0.90 for CRS prediction; advance warning up to 72 h before clinical onset
ML on patient-reported outcomes	Psychosocial adverse events (depression, anxiety)	Leukemia/lymphoma (various ICI therapies)	Identifies high-risk patient subgroups; sensitivity approximately 70–80% across reported cohorts
Wearable sensor ML (vitals, activity)	Life-threatening immune-related events	Solid and hematologic cancers (ICI/CAR-T patients)	Accuracy 70–90% for early detection of life-threatening adverse events in retrospective monitoring studies

*RF* random forest, *XGBoost* gradient boosting, *AE* adverse event, *ICANS* immune effector neurotoxicity syndrome

### Translational potential in emerging heme therapies

Novel treatments for blood cancers have great potential, and the use of AI can improve their predictive power, delivery, and personalisation.

#### New and emerging therapies

1. *Cellular Therapies*: CAR-T cell and engineered T-cell receptor (TCR) therapies are reshaping the landscape for blood cancers (lymphoma, leukaemia, and multiple myeloma) [6].
2. *Bispecific Antibodies & ADCs*: Bispecific antibodies, such as Lynozyfic (approved in 2025), and antibody-drug conjugates (ADCs) are advancing targeted immunotherapy, enabling precise targeting of cancer cells [12, 16].
3. *Advanced Nanomedicine*: Recent progress involves ligand-directed nanoparticles that specifically target drugs to bone marrow tumour cells, increasing bioavailability and reducing systemic toxicity (especially in leukaemia) [17, 26].

#### Translational Role of Artificial Intelligence

1. *Biomarker Discovery*: Biomarkers enabled by machine learning and big data improve disease classification and treatment, enabling precise medical care [11, 29].
2. *Personalisation & Customisation*: By combining clinical, genomic, and bioinformatic information, AI tailors antibody, cellular, and nanomedicine therapies to each patient’s profile, providing a more personalised approach to treating blood cancers [29, 30].

#### Challenges and Future Outlook

1. *Translational Barriers*: Key challenges are biological variability, immune response to novel delivery methods, and scaling complex therapies. AI assists in addressing these difficulties by providing enhanced prediction and targeted patient and disease care [19, 29].
2. *Personalised Nanomedicine*: The use of AI and advanced diagnostics is poised to become a key factor in the safe, effective, and scalable treatment of blood cancers [26, 30].

## Integrating AI for personalised immunotherapy

Today, AI in cancer immunotherapy can become true precision medicine by combining diverse data types, such as genomics, transcriptomics, imaging, pathology, and clinical records. This approach helps capture the complex interactions between tumours and the immune system. Using AI for tumour microenvironment profiling and multimodal data can uncover new biomarkers and predict treatment response. Both strategies can support patient-focused therapies, improve treatment decisions, and help move immunotherapy into routine practice.

### Novel biomarker discovery

AI in digital pathology uses H&E-stained whole slide images to study cancer tissues and the immune cells within them. This is done by analysing patterns such as tumour-infiltrating lymphocytes (TILs) and features of the tumour microenvironment. AI can identify immune biomarkers beyond standard tests like PD-L1, MSI, or TMB. With advanced tools such as multiplex immunohistochemistry and multi-omics integration, AI can reveal new tumor–immune interactions and support the discovery of more accurate biomarkers to predict immunotherapy response and patient survival [11].

Highlights from ASCO 2025 showcase how AI in digital pathology can provide key molecular features, for example, HER2, BRCA, and MSI, to generate prognostic scores like CAPAI by combining pathology with clinical data to enable it to predict treatment response or risk using routine H&E slides and circulating tumour DNA. These advances have improved biomarker discovery well beyond the capabilities of traditional assays, thus offering powerful tools for precision oncology [17]. Artificial intelligence and digital biomarkers for precise oncology are increasingly used to identify precise biomarkers in immuno-oncology. AI enables the detection of spatial, contextual, and subtle morphological patterns in tumour tissues that can predict immune response and treatment outcomes. This work reflects growing adoption and scientific confidence in AI-driven biomarker discovery for precision cancer care [15].

### TME profiling

**Fig. 2:**
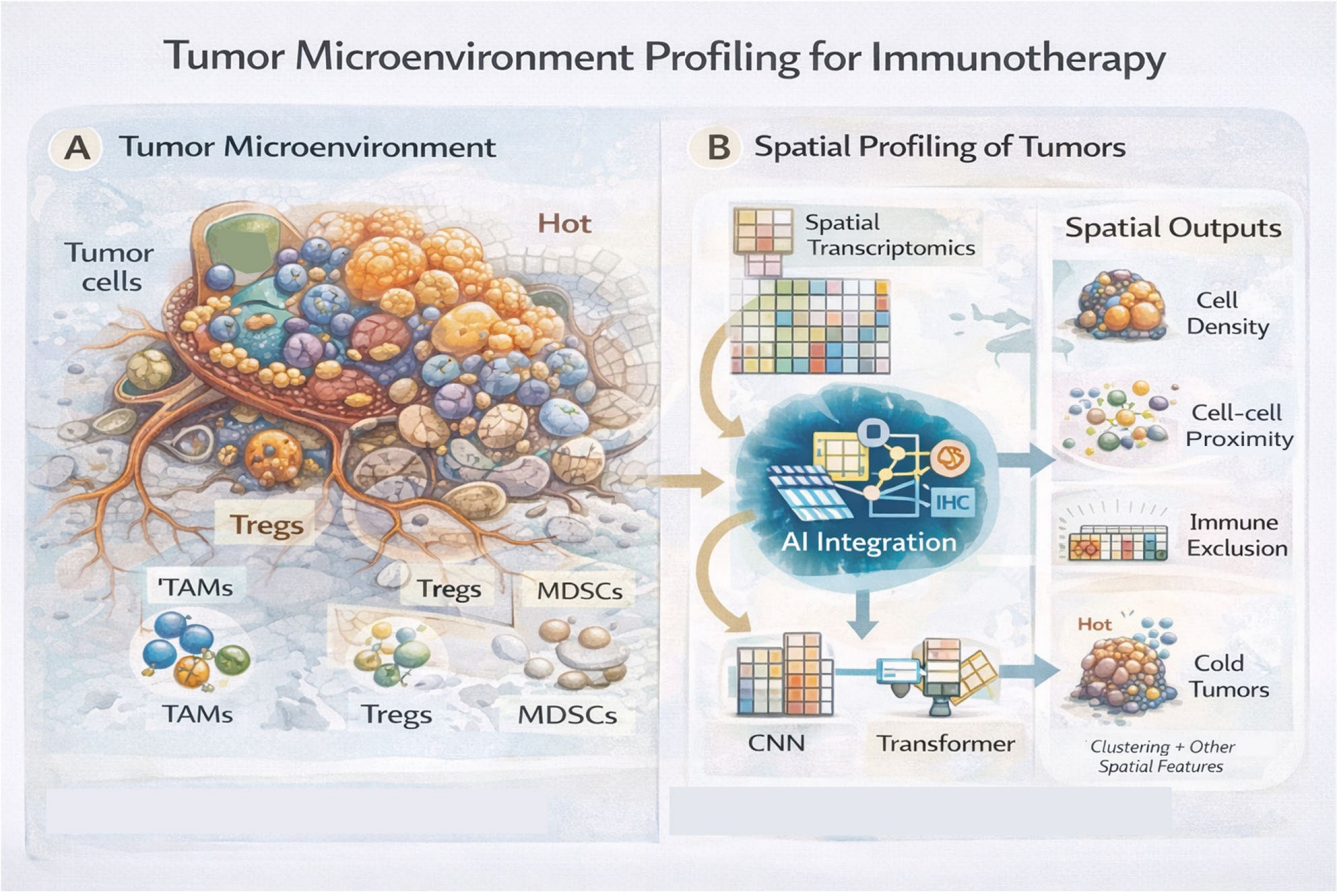
AI-based tumour microenvironment (TME) profiling for immunotherapy response prediction. **A** The cellular architecture of the TME, depicting the spatial distribution of tumour cells alongside key immunosuppressive populations, tumour-associated macrophages (TAMs), regulatory T cells (Tregs), and myeloid-derived suppressor cells (MDSCs), and the distinction between immune-hot (inflamed) and immune-cold (excluded) tumour phenotypes. **B** The AI-based spatial profiling pipeline, in which spatial transcriptomics and immunohistochemistry (IHC) data are processed through convolutional neural networks (CNNs) and transformer-based architectures to generate clinically actionable spatial outputs: cell density, cell-cell proximity, immune exclusion topology, and tumour immune phenotype classification. TAMs, tumour-associated macrophages; Tregs, regulatory T cells; MDSCs, myeloid-derived suppressor cells; CNN, convolutional neural network; IHC, immunohistochemistry

AI-based tumour microenvironment profiling illustrates the spatial and cellular complexity of tumours across histopathology, single-cell sequencing, and spatial transcriptomics. These methods also reveal patterns of immune-cell infiltration and checkpoint-molecule expression, enabling classification of hot and cold tumours. It therefore enables better prediction of immunotherapy responses and informs strategies to overcome resistance, establishing TME profiling as the basis for personalised cancer immunotherapy [26]. Figure 2 illustrates the AI-based tumour microenvironment profiling pipeline and its spatial outputs.

Artificial intelligence-based tumour microenvironment (TME) profiling is transforming personalised immunotherapy by enabling multidimensional characterisation of tumour immune and stromal environments, with clear, direct relevance to treatment outcome prediction and optimisation [26]. Designed AI algorithms examine histopathology, transcriptomics, and radiomics to accurately determine the composition, localisation, spatial allocation, and cellular interactions of the TME, thereby improving the response to immunotherapy. Experts have demonstrated the ability to predict patient prognosis by extracting spatially resolved TME signatures from standard pathology slides, in which population- and cell-level interactions predict patient outcome through immunotherapy (e.g., AUROC in independent cohorts can reach 0.75). AI has the potential to measure immune infiltrates (e.g., tumour-infiltrating lymphocytes), predict biomarker expression (PD-L1, TMB, MSI), and identify immune microenvironment features inaccessible to traditional pathology methods, providing real-time, noninvasive, and high-throughput disease assessment to guide therapy choice.TME profiling is a fast-evolving AI-powered cornerstone of individualised immunotherapy, enabling more profound immune characterisation and real-time clinical decision-making [26].

### Multi-modal integration

Multi-modal integration with AI incorporates genomics, transcriptomics, imaging, pathology, and clinical data to reveal trends in tumour-immune interactions. These models enable biomarker discovery, prediction of treatment response, and adaptive treatment planning by extracting information from multiple data types. This system-wide strategy also allows greater differentiation of patients and takes the next step forward towards providing real immunotherapy [29]. Multimodal integration in AI uses multiple types of data to improve prediction, assess treatment response, and differentiate immunotherapy protocols for specific patients: genomics, medical imaging, digital pathology, and clinical records. Transformers and Graph Neural Networks (GNNs) are advanced deep learning architectures that combine diverse data (radiology, pathology, genomics, and clinical metrics) to reveal patterns when predicting the survival of cancer patients undergoing immunotherapy [29, 30].

Multi-modal models have always outperformed unimodal methods in forecasting treatment response, risk of relapse, and survival, and have been demonstrated to have an area under the curve (AUC) that is significant in predicting survival and therapy choice, ranging between 0.80 and 0.91 [30]. A combination of longitudinal and routine diagnostic data, such as CT scans, blood tests, medication history, and genome assays, enables this in real-time, regardless of cancer type. Multi-modal integration can characterise tumours holistically, accounting for the spatial, molecular, and clinical heterogeneity necessary to personalise immunotherapy. It makes stronger predictions about the result and is more effective than the traditional single-marker biomarkers of TMB or PD-L1 in isolation, with the goal. It accelerates the transition from precision medicine to daily oncological practice. AI-based multimodal integration will rapidly promote the development of personalised immunotherapy with enhanced predictive capabilities and more customised treatment plans for cancer patients.

## Limitations, validation, and interpretability

### Data-related challenges

The use of AI in blood cancers has been significantly hindered due to issues with data quality, bias, and sample size [19]. These issues affect the accuracy, reliability, safety, and fairness of AI in cancer immunotherapy and in predicting therapy response.

#### Data and quality challenges

To be accurate and reliable, AI models require massive, well-crafted datasets of genomic, clinical, and imaging data [19]. Poor-quality, inconsistent data labelling (e.g., differences in sample preparation, imaging, and flow cytometry) reduces model performance and reproducibility [14, 19]. In haematology, where rare cancer cases are common, high-quality whole-slide images and annotated samples are often unavailable, which limits the application of deep learning models [29]. Organising data across centres remains a constant barrier due to varying standards and data silos [18, 28].

#### Bias and its impacts

Algorithmic bias arises from skewed or unbalanced data, leading to poorly applied AI models to diverse populations and exacerbating healthcare inequities [19]. Haematological cancer risk AI models are biased and do not generalise well on non-demographically representative datasets without external validation, leading to unequal diagnostic accuracy across groups [19, 29].

#### Data size and limitation

Haematological AI research is usually based on small, limited datasets because some blood cancers are rare and data sharing is restricted, leading to overfitting, poor calibration, and decreased model robustness across or to future datasets [29]. The labour further constrains the scope of growth and the variety of available datasets- and the expertise-intensive nature of expert annotation, especially for pathology images.

### Prospective validation and reproducibility gaps

Artificial intelligence shows promise for predicting immunotherapy response, but critical validation and reproducibility gaps severely limit its clinical translation [18]. Only 1.88% of AI models developed for cancer diagnosis reach prospective clinical use, with most confined to retrospective analyses [18, 20].

#### Prospective validation crisis and reproducibility challenges

Most AI immunotherapy models lack prospective validation through randomised controlled trials [18, 20]. When tested prospectively, AI performance often degrades more than in retrospective results, external validation frequently shows performance drops from AUROCs of 0.80 + to the 0.66–0.70 range [18]. Less than 50% of immunotherapy AI studies include external validation cohorts. This validation deficit represents the most pressing barrier to clinical implementation.

Moreover, research on AI in oncology faces challenges in data standardisation due to inconsistent imaging protocols, tumour segmentation, and data collection procedures, making it difficult to reproduce the model. Prejudice in datasets also makes the outcomes more difficult, since the majority of the participants in the studies are White, and not many of them were racial. Academic institutions commonly provide training data that does not represent minority and low-income groups and therefore cannot be generalised to these groups. Additionally, inadequate methodological descriptions and disjointed algorithm development decrease the reproducibility of most studies.

#### Barriers to implementation and the path forward

There are various challenges to implementing AI in cancer immunotherapy, such as ineffective regulatory frameworks that prioritise technical accuracy over clinical use. Other challenges include the inability to integrate AI into current workflows, the lack of infrastructure, and the rapid pace of AI innovation, which make steady implementation challenging [20, 21]. The state of the art requires standardised protocols, multi-institutional clinical trials, multi-representative training datasets, and more powerful regulatory systems to proceed. Interdisciplinary collaboration and high-level validation criteria are needed to ensure reproducibility and realise the potential of AI to deliver personalised cancer immunotherapy [28].

### Interpretability, transparency, and clinical trust

Many AI models are black boxes, which remains a significant obstacle to their application in cancer immunotherapy, where decisions can affect patient survival [23]. Most systems are not interpretable, even though they have high predictive accuracy, which has limited their adoption by clinicians. Physicians find it hard to comprehend AI-generated recommendations because integrating them into the clinical workflow is challenging [20, 23]. Explainable AI (XAI) methods such as SHAP and LIME help address this issue by clarifying how features influence predictions. Studies suggest that transparency improves physician confidence in AI-generated recommendations [23]. For instance, SHAP has highlighted key biomarkers, such as the neutrophil-to-lymphocyte ratio, as strong predictors of immunotherapy outcomes in NSCLC patients, helping clinicians verify AI insights with medical knowledge [17]. More recent studies have also shown that explainable AI, when used with multimodal models and pathology, can enhance transparency and clinical utility, allowing quantitative assessment of model behaviour and the identification of embedded bias [22, 26].

Nevertheless, the trust in AI is complicated and relative. Physicians’ acceptance depends on the type of cancer, the complexity of treatment, and agreement with conventional care, and patients are still more inclined to follow a physician’s decision than AI-based suggestions. Transparency should not be limited to algorithm descriptions alone; it should also cover information about the training data, biases, and limitations. Nevertheless, the majority of medical AI applications are low in openness and exhibit 22.7–60.9% transparency. More stringent transparency standards, user-friendly clinician interfaces, and ethically and collaboratively oriented applications are needed to achieve reliable AI in immunotherapy. Lastly, to promote patient-centred, interpretable, and safe cancer care, AI cannot replace human knowledge; rather, it should complement it.

## Future perspectives and clinical translation

### Next-Gen AI methods (Federated Learning, Foundation Models)

Federated Learning (FL) enables multiple healthcare institutions to jointly train AI models without violating patient privacy, with raw data stored at each local site. FL avoids having all the data located in a single location and instead uses distributed computation to construct shared models, minimising the problems of data scarcity and population bias prevalent in traditional AI systems [28]. FL-based models have performed remarkably well in cancer research, achieving AUCs of 0.710–0.869 to predict gastric cancer recurrence across four centres and 98.9% accuracy in breast cancer classification [28, 29]. It should be noted, though, that these findings are mainly based on retrospective or limited-cohort studies and need to be confirmed across a variety of populations before clinical applicability can be advised. FL frameworks help predict treatment responses and adverse events in immunotherapy using real-world, multicenter datasets [29].

The Foundation Models exploit large-scale self-supervised pretraining across millions of cancer samples to create generalisable representations for diverse oncology tasks [30]. Specialised models such as MUSK, which combine pathology images with clinical data from 50 million images, are more effective at predicting immunotherapy outcomes than traditional methods. Equally, the Threads model, which is trained on more than 47,000 tissue sections and paired with genomic information, has demonstrated generalizability across 54 oncology tasks, including mutation detection and treatment response prediction. Vision Transformers that can capture spatial tumour heterogeneity achieve 96–99% accuracy in classification tasks important for immunotherapy decisions [29, 30].

ChatGPT-4 and larger Language Models (LLMs) like OncoGPT and CancerLLM are highly performing models, achieving approximately 75% accuracy in answering immuno-oncology questions and predicting drug sensitivity. The C2S-Scale foundation model developed by Google has even generated new hypotheses in immunotherapy, which were later experimentally confirmed [30]. The combination of various types of data, imaging, genomics, and clinical information, with transformer-based architectures further enhances the accuracy and robustness of predictions [29, 30]. Combined, these next-generation AI systems address the long-term problems of data scarcity, privacy, and generalizability, enabling scalable, personalised immunotherapy for cancer [28, 30].

### Emerging data streams

Spatial omics technologies map tumour microenvironmental molecules and maintain tissue architecture, thereby clarifying how immune cells communicate spatially with tumours, in contrast to conventional bulk sequencing, which obscures heterogeneity [26]. Spatial transcriptomics and proteomics record cell-to-cell interactions that are important in immunotherapy response [26]. The AUC of spatial multi-omics for predicting immunotherapy outcomes in NSCLC is 0.838, which is much higher than that of traditional biomarkers. AI-based analysis can detect new spatial resistance features and virtual biomarkers, such as restrictive extracellular matrices between tumours and immune aggregates, in non-responders [26, 29].

Digital twins (DTs) are virtual patient models that combine genomics, imaging, clinical data, and real-time measurements to simulate disease progression and individualise immunotherapy plans [28]. Theranostic digital twins are effective at predicting checkpoint inhibitor and PD-L1 treatment response, which, in turn, informs pre-treatment outcomes. In silico experiments with DTs save time on patient recruitment and pre-identify those who will respond to immunotherapy before clinical injection. With AI-controlled wearables, one can continuously monitor cancer patients without being invasive, tracking vital signs, activity, and biomarkers during immunotherapy. Wearables using machine learning algorithms achieve 70–90% accuracy in identifying immune-therapy complications and in identifying life-threatening adverse events before they manifest in the clinical setting. Wearable data, which are part of digital twins, enable dynamic feedback systems that adjust treatment regimens in response to real-time physiological data [27]. Includes critiques such as data privacy, gaps in standardisation, limitations in sensor accuracy, and barriers to integration in healthcare. These convergent streams of data represent a paradigm shift toward holistic, spatially aware, continuously monitored, AI-driven, personalised cancer immunotherapy.

### Toward clinical-grade AI and regulatory pathways

The FDA considers AI to be a risk-based Software-as-a-Medical-Device (SaMD) [21]. The January 2025 draft guidance proposes a seven-step credibility assessment model that focuses on validation, transparency, and lifecycle monitoring, Oncology AI Program (launched 2023). The Oncology AI Program offers a special review of AI-enabled cancer drug development to the FDA [20]. Only 1.88% of AI oncology tools achieve clinical deployment [20]. FDA needs prospective randomised trials that demonstrate better patient outcomes, but most systems lack real-world validation evidence [18, 20]. The existing requirements demand excellence compared to traditional methods, which most AI models cannot achieve [18]. Of the 71 AI devices approved by the FDA as oncology devices, 54.9% are radiology devices and 19.7% are pathology devices, with more than 80% of these devices being diagnostic [21]. Some recent advancements include multi-organ cancer detection by Paige (2024) and autonomous AI agents with 87.2% clinical accuracy [21].

Most studies base their findings on retrospective data rather than validating them prospectively and multicentrically in a varied population [18]. Real-world clinical environments have shown significant performance degradation due to external validation [18, 19]. Existing substantial equivalence pathways might not be adequate for assessing new AI architectures [21]. AI implementation needs to be explainable; there must be EHR integration, and frameworks for human-AI collaboration, not autonomous decision-making [21, 23]. It has resistance to workflow and to untrained clinicians, which impede adoption. The regulatory models must enable an AI system to continue learning by providing Algorithm Change Protocols. The work of international harmonisation aims to represent a large population and minimise bias. To achieve clinical-grade AI, a transition of technical measures into clinical benefit and improved patient outcomes is needed.

## Conclusion

AI has emerged as a game changer in cancer immunotherapy, specifically in addressing the long-standing issue of inter-patient variability in treatment response. The AI-based models provide a more detailed description of tumour biology and the tumour microenvironment by incorporating high-dimensional data (genomics, transcriptomics, proteomics, radiomics, and clinical records) into the model. Machine learning and deep learning approaches have become more helpful for predicting response to immune checkpoint inhibitors, identifying new composite biomarkers, and patient stratification, and are not reliant on traditional one-parameter biomarkers, such as PD-L1 expression or tumour mutational burden. It is noteworthy that AI will also support dynamic and longitudinal assessment and enable treatment decisions to keep pace with tumour adaptations and immune remodelling. All of the mentioned developments make AI a key to truly personalised immunotherapy, enabling high response rates, reducing unjustified toxicity, and improving clinical decision-making.

Although it has promise, AI as a clinical translation of immunotherapy is stalled by issues of data quality, model understanding, reproducibility, and regulatory acceptability. It will require robust prospective validation across other patient groups to ensure the results are generalisable and to avoid algorithmic bias. Moreover, the data-generation protocols and transparent reporting models should be standardised to reduce the gap between the computational and clinical uptake. The direction this will take in the future will be marked by high levels of interdisciplinary collaboration among oncologists, immunologists, data scientists, bioinformaticians, and regulatory bodies. Such collaboration is necessary not only to refine predictive models but also to implement ethical, explainable, and patient-centric principles in AI-based tools. One possible way AI can be used to treat patients is by making it a stable, meaningful tool for precision cancer immunotherapy, part of a treatment approach that can help individuals coordinate their efforts.

## Data Availability

This manuscript is a review article; no new datasets were generated or analysed. All data and information supporting the conclusions are derived from previously published, publicly available sources and are fully cited in the References. Where applicable, readers may consult the original cited studies to access underlying datasets. Additional materials or clarifications can be obtained from the corresponding author, Eloghosa Nosa-Ihaza (eloghosa127@gmail.com), upon reasonable request.
